# Imposed glutathione-mediated redox switch modulates the tobacco wound-induced protein kinase and salicylic acid-induced protein kinase activation state and impacts on defence against *Pseudomonas syringae*

**DOI:** 10.1093/jxb/eru546

**Published:** 2015-01-26

**Authors:** Sanja Matern, Tatjana Peskan-Berghoefer, Roland Gromes, Rebecca Vazquez Kiesel, Thomas Rausch

**Affiliations:** ^1^Centre for Organismal Studies Heidelberg, Department of Plant Molecular Physiology, Heidelberg University, 69120 Heidelberg, Germany; ^2^The Hartmut Hoffmann-Berling International Graduate School of Molecular and Cellular Biology (HBIGS), Heidelberg University, 69120 Heidelberg, Germany

**Keywords:** Glutathione, hypersensitive response, priming, *Pseudomonas syringae*, redox state, salicylic acid, SIPK/WIPK.

## Abstract

The role of the redox-active tripeptide glutathione in plant defence against pathogens has been studied extensively; however, the impact of changes in cellular glutathione redox potential on signalling processes during defence reactions has remained elusive. This study explored the impact of elevated glutathione content on the cytosolic redox potential and on early defence signalling at the level of mitogen-activated protein kinases (MAPKs), as well as on subsequent defence reactions, including changes in salicylic acid (SA) content, pathogenesis-related gene expression, callose depositions, and the hypersensitive response. Wild-type (WT) *Nicotiana tabacum* L. and transgenic high-glutathione lines (HGL) were transformed with the cytosol-targeted sensor GRX1-roGFP2 to monitor the cytosolic redox state. Surprisingly, HGLs displayed an oxidative shift in their cytosolic redox potential and an activation of the tobacco MAPKs wound-induced protein kinase (WIPK) and SA-induced protein kinase (SIPK). This activation occurred in the absence of any change in free SA content, but was accompanied by constitutively increased expression of several defence genes. Similarly, rapid activation of MAPKs could be induced in WT tobacco by exposure to either reduced or oxidized glutathione. When HGL plants were challenged with adapted or non-adapted *Pseudomonas syringae* pathovars, the cytosolic redox shift was further amplified and the defence response was markedly increased, showing a priming effect for SA and callose; however, the initial and transient hyperactivation of MAPK signalling was attenuated in HGLs. The results suggest that, in tobacco, MAPK and SA signalling may operate independently, both possibly being modulated by the glutathione redox potential. Possible mechanisms for redox-mediated MAPK activation are discussed.

## Highlight

Transgenically imposed high glutathione, uncoupled from cellular controls, causes an oxidative shift in cytosolic redox potential, activates tobacco MAPKs, and primes immune responses to adapted and non-adapted *Pseudomonas syringae* pathogens.

## Introduction

In higher plants, the first line of defence against pathogens is triggered by pathogen/microbe-associated molecular patterns (P/MAMPs), causing activation of pattern-triggered immunity (PTI). In the course of evolution, micro-organisms have evolved effector proteins to cope with PTI. If suppression of defence barriers by microbe effectors has been successful, pathogens will cause disease, classifying them as *adapted* pathogens for a given host plant. In response, plants have evolved effector-triggered immunity as a second line of defence, which is highly pathogen or pathovar specific and is mediated by recognition of pathogen effector(s) via specialized resistance proteins (Chisholm *et al.*, 2006; Pieterse *et al.*, 2009). Signalling triggered by immunity receptors is mediated by reactive oxygen species (ROS) and mitogen-activated protein kinases (MAPKs), resulting in transcriptional reprograming, activation of defence proteins and metabolites, cell-wall reinforcement, and programmed cell death (Boller and Felix, 2009). Besides mounting an all-out defence against infection, plants are also able to acquire a *primed* state that enables them to respond to biotic or abiotic stress faster and more efficiently (Conrath, 2011).

Previous studies have explored the role of the tripeptide glutathione in plant immunity, motivated by its multiple functions as an intracellular redox buffer (Mou *et al.*, 2003), as a substrate for glutathionylation of target proteins (Zaffagnini *et al.*, 2012), and because of its involvement in the biosynthesis of sulfur-containing plant defence compounds (Geu-Flores *et al.*, 2011; Su *et al.*, 2011). Lowering the overall cellular glutathione content has a profound effect on plant defence against pathogens. Thus, glutathione-deficient *Arabidopsis thaliana* mutants showed increased susceptibility to several pathogens: the *pad2* mutant displayed an increased susceptibility to the bacterium *Pseudomonas syringae*, to the oomycete *Phytophtora brassicae* (Parisy *et al.*, 2007; Dubreuil-Maurizi *et al.*, 2011), and to the fungus *Alternaria brassicicola* (van Wees *et al.*, 2003). Furthermore, the mutants *cad2* and *rax1* showed increased susceptibility to an avirulent *P. syringae* strain, concomitant with decreased transcript levels for genes involved in plant resistance to pathogens (Ball *et al.*, 2004). All three mutants are defective in γ-glutamylcysteine ligase (GCL), the key enzyme for glutathione biosynthesis (Hothorn *et al.*, 2006). While an impairment of defence against pathogens was consistently observed, the underlying molecular mechanism(s) are of complex nature, as in the Brassicaceae glutathione is—besides its role as a redox buffer—involved in the biosynthesis of glucosinolates and the phytoalexin camalexin (Roetschi *et al.*, 2001; Schlaeppi *et al.*, 2008).

The cellular redox state was also shown to be of crucial importance for the salicylic acid (SA)–NON-EXPRESSOR OF PR GENES 1 (NPR1) signalling pathway. In the cytosol, NPR1 protein monomers are released from large disulphide bridge-linked complexes and are subsequently translocated to the nucleus, where they associate with transcription factors to activate defence genes (Mou *et al.*, 2003). The release of NPR1 monomers required thioredoxins, whereas *S*-nitrosoglutathione facilitated NPR1 oligomerization (Tada *et al.*, 2008). Recently, Han *et al.* (2013) demonstrated that, upon transfer from a high-CO_2_ environment (blockage of photorespiration) to ambient air, the catalase-deficient *Arabidopsis* mutant *cat2* displayed a 2-fold increase in total glutathione content, with its glutathione pool being more than 50% oxidized. This shift in glutathione oxidation state was accompanied by a strong increase in free SA and its glucoside SAG (Chaouch *et al.*, 2010), concomitant with restricted propagation of the virulent pathogen *P. syringae* DC3000 during the early infection stage (Han *et al.*, 2013). Conversely, in an *Arabidopsis* mutant for cytosolic glutathione reductase (*gr1*), which also displayed an increased oxidation state of its glutathione pool (although less pronounced than in the *cat2* mutant; Mhamdi *et al.*, 2010), total glutathione content was not significantly increased (Marty *et al.*, 2009), and SA content was rather decreased compared with that of the wild type (WT). These observations suggested that both total glutathione content and degree of oxidation may impinge on SA accumulation.

Besides SA-induced NPR1-dependent signalling, MAPKs can also induce defence gene expression in a SA/NPR1-independent manner (Tsuda *et al.*, 2013). While the role of ROS and redox change appear to be important for the P/MAMP-mediated activation of the MAPK cascade (Liu *et al.*, 2007; Xu *et al.*, 2014b), the molecular mechanism(s) that trigger MAPK signalling may depend on host species and elicitor type (Segonzac *et al.*, 2011; Xu *et al.*, 2014a). Hints for a possible direct redox regulation of MAPKs come from mammalian studies including redox-sensitive cysteine of MAPKs and MAPK phosphatase (Kim *et al.*, 2012; Bassi *et al.*, 2014).

Based on these results and the previously reported increase of glutathione during pathogen attack under physiological conditions (Vanacker *et al.*, 2000; Parisy *et al.*, 2007), the present study addressed the following questions: (i) To what extent does an increased glutathione content (induced transgenically or pharmacologically) affect the cytosolic redox state? (ii) Does an increase in glutathione content and/or change in redox state also impact on MAPK signalling, SA accumulation, and pathogenesis-related (PR) gene induction in the absence of pathogen challenge? (iii) Which defence responses are targeted by glutathione upon *P. syringae* infection?

To answer these questions, the cytosolic glutathione redox state was monitored with a redox sensor (GRX1-roGFP2) in a genetic background of WT and transgenic lines, expressing the bifunctional glutathione biosynthetic enzyme from *Streptococcus thermophilus* (StGCL-GS combines the activities of γ-glutamylcysteine ligase and glutathione synthetase; Liedschulte *et al.*, 2010). The results obtained from this transgenic approach with high-glutathione lines (HGLs)—and further corroborated by pulse feeding of reduced glutathione (GSH) or the oxidized form (GSSG) to leaves of WT tobacco—demonstrated a direct effect of the cytosolic glutathione redox state on the activation state of the MAPKs wound-induced protein kinase (WIPK) and SA-induced protein kinase (SIPK), while possible mechanisms for a post-translational redox modulation of the MAPK cascade are discussed. Furthermore, HGLs displayed improved defence against adapted and non-adapted strains of *P. syringae* at the level of SA accumulation, PR gene expression, callose deposition, and the hypersensitive response (HR).

## Materials and methods

### Plant material, growth conditions, and generation of transgenic lines

Seeds of WT (*Nicotiana tabacum* Samsun NN) and transgenic lines expressing the bacterial bifunctional enzyme (StGCL-GS) under the control of the constitutive cauliflower mosaic virus 35S promoter (Liedschulte *et al.*, 2010) were sterilized and placed on MS agar for germination. Transgenesis was confirmed by selection on kanamycin. After selection, plants were transferred to soil and grown in the greenhouse for 6 weeks under long-day conditions (16h light per day, temperature 24±2 °C). For stable expression of reduction-oxidation sensitive green fluorescent protein (roGFP2), the cassette of GRX1:roGFP2 plus ubiquitin promoter (present in the pBinAR vector; kindly provided by A. Meyer, Bonn, Germany) was amplified with primers cytRoGFP_F and cytRoGFP_R (listed in Supplementary Table S1 at *JXB* online) and inserted into plasmid vector pSS02 to allow selection for hygromycin resistance. Subsequently, this construct was mobilized into *Agrobacterium tumefaciens* strain C58C1 and used for stable transformation of tobacco leaf discs (Gallois and Marinho, 1995) in a WT background and four different StGCL-GS lines with five to six individual plants per line that displayed different degrees of glutathione accumulation.

### Bacterial growth and infection protocol

For infection experiments, *P. syringae* strains were grown in Luria–Bertani broth overnight, washed, and resuspended in 10mM MgCl_2_ to an optical density (OD_600_) of 0.5, corresponding to approximately 5×10^8^ colony-forming units (CFU) ml^–1^. Aliquots of appropriate bacterial dilution (50 µl) were infiltrated into tobacco leaves using a 10ml syringe without needle applied to the abaxial side of the leaf (Thilmony *et al.*, 1995). To monitor bacterial propagation in the leaf, two leaf discs were excised (diameter 1cm^2^) from each plant and macerated in 1ml of 10mM MgCl_2_ (Katagiri *et al.*, 2002). Bacterial number was counted after plating appropriate serial dilutions on Luria–Bertani agar plates supplemented with 100mg l^–1^ of rifampicin or ampicillin. Mock treatment included the same treatment with 10mM MgCl_2_. Plant pathogenic bacteria including *P. syringae* pv. *tabaci* ATCC 11527 (*Pst*), *P. syringae* pv. *maculicola* ATCC 33190 (*Psm*) and *P. syringae* pv. *syringae* ATCC 19310 (*Pss*) were purchased from the German Collection of Microorganisms and Cell Cultures (DMSZ, Braunschweig, Germany). *P. syringae* pv. *tabaci* ATCC 11528 was obtained from the Sainsbury Laboratory, UK.

### Glutathione measurement

Thiols were extracted from 30mg of plant tissue in the presence of dithiothreitol (DTT; for total glutathione) or *N*-ethylmaleimide (NEM; for oxidized glutathione) and derivatized with monobromobimane (Sigma) as described previously (Fey *et al.*, 2005). Glutathione was measured by reverse-phase high-performance liquid chromatography (HPLC) according to Liedschulte *et al.* (2010). GSH was determined by subtracting GSSG from total glutathione.

### *In vivo* imaging of the cellular redox state by confocal laser-scanning microscopy and ratiometric analysis

Images of epidermal cells from plants stably transformed with the GRX-roGFP2 sensor were taken with an LSM510META (Carl Zeiss MicroImaging, Germany), using 405 and 488nm excitation wavelengths as described by Schwarzlaender *et al.* (2008). Leaves were infiltrated with 100mM DTT or 50mM H_2_O_2_ for calibration of the probe. In preliminary experiments, it was confirmed that these concentrations were sufficient for full reduction or oxidation of the probe. To monitor the cellular redox state (with or without infection), leaf areas were mock infiltrated with sterile distilled water or bacterial suspension, and, subsequent microscopic analysis was done avoiding the area of syringe contact. Fluorescence ratio analysis, including the degree of oxidation and calculation of redox potential (Meyer *et al.*, 2007), was carried out via the MatLab (The MathWorks, USA) analysis suite, kindly provided by A. Meyer (Bonn, Germany).

### Immunoblot analysis of MAPKs and activation status of SIPK/WIPK

The amounts of activated MAPK proteins (SIPK and WIPK) were determined by immunoblot analysis with anti-pTEpY (anti-ERK1/2) antibody, purchased from Cell Signalling. Total SIPK protein was detected with anti-AtMPK6 antibody (Sigma) and total WIPK protein with anti-AtMPK3 antibody (Sigma). Total protein was extracted using the protocol of Segonzac *et al.* (2011). Different amounts of protein were loaded per lane depending on the experimental setup (see Results).

### Determination of free and total SA

The method was adapted from DeFraia *et al.* (2008), using *Acinetobacter* sp. ADPWH _*lux* strain (Huang *et al.*, 2005, 2006). In brief, 100mg of tissue was frozen in liquid nitrogen and thoroughly ground, and 250 µl of acetate buffer (0.1M, pH 5.6) was added. Samples were then mixed and centrifuged for 15min at 16 000*g*. Half of the supernatant was stored on ice for determination of free SA, whereas the other half was incubated at 37 °C for 2h with 10U of β-glucosidase (Sigma) for determination of total SA, consisting largely of free SA and its glucoside (SAG). *Acinetobacter* sp. ADPWH_lux strain was diluted to an OD_600_ of 0.35 and incubated with 20 µl of plant crude extract for 2h in a microtitre plate. Luminescence was detected on a plate reader (Fluostar Omega; BMG LabTech, Germany). Standards contained known concentrations of SA.

### Determination of electrolyte leakage by conductivity measurement

The protocol was adapted from Torres *et al.* (2002). At 24h post-infection (hpi), 10 leaf discs (diameter 1cm) were collected from infected area, washed with double-distilled water, and placed in a tube with 20ml water. In mock treatments, plants were injected with 10mM MgCl_2_. Conductivity was measured with a conductivity meter (Schott, Germany) and expressed as µS cm^–1^.

### Quantitative polymerase chain reaction analysis

Total RNA was extracted using a Gene Matrix Universal RNA Purification kit (EurX) and reverse transcribed with a SuperScript III First-Strand Synthesis System from Invitrogen. Quantitative polymerase chain reaction was performed using the following conditions: 95 °C for 6min, followed by 40 cycles of 95 °C 30 s, 60 °C 20 s, and 72 °C for 30 s. All primer sequences used in this study are listed in Supplementary Table S1. Ribosomal protein L25 was used as reference gene for the gene expression normalization using the ΔΔ*C*_t_ method. For independent confirmation of the results, ubiquitin and elongation factor 1α were used as additional reference genes.

### Callose detection

For visualization of callose, leaf discs were cut from inoculated regions and incubated with ethanol:acetic acid solution (1:3, v/v) overnight to completely remove leaf pigments. After rehydration, the leaf discs were stained with 0.01% aniline blue in 150mM K_2_HPO_4_, pH 9 (Millet *et al.*, 2010). Stained tissue was examined using ultraviolet epifluorescence (DAPI filter; Leika), avoiding areas close to wounds from inoculation or leaf sample excision. Callose deposits were counted per 1.5mm^2^ microscopy field, using ImageJ software.

### Determination of H_2_O_2_

H_2_O_2_ was determined according to Wi *et al.* (2012), based on the oxidation of dichlorodihydrofluorescein diacetate (DCFH-DA; Sigma). In brief, after homogenization of tissue with 10mM Tris/HCl buffer (pH 7.2), the supernatant was incubated with DCFH-DA at room temperature for 10min in the dark. DCF fluorescence was detected using a plate reader (Fluostar Omega; BMG LabTech) at an excitation wavelength of 485nm and an emission wavelength of 525nm. Relative fluorescence was normalized to milligrams of protein. For 3,3’-diaminobenzidine staining, leaf discs were cut at 24 hpi and stained in 1mg ml^–1^ of 3,3’-diaminobenzidine for 6h. The leaf discs were then destained with 96 % ethanol and rehydrated in 50% glycerol. Samples were visualized by bright-field microscopy.

### Statistical analysis

Student’s *t*-test was used to analyse statistically significant differences between transgenic lines and WT, and between different treatments. Significant differences were indicated for *P*≤0.05 and *P*≤0.01.

## Results

### Glutathione redox potential of HGLs reveals oxidized patches in the cytosol

HGLs with constitutive expression of a bacterial bifunctional enzyme for glutathione synthesis (StGCL-GS), covering a broad range of glutathione accumulation (i.e. from about 3- to more than 10-fold increase compared with WT) have been described previously by Liedschulte *et al.* (2010). For the present study, two types of HGLs were selected: lines with cytosolically localized bacterial enzyme (cyt) and lines with StGCL-GS targeted to the plastids (pls). Note that the HGLs used in this study displayed 2- to 5-fold higher glutathione content compared with WT, this increase being in the same range as reported for the pathogen-induced glutathione increase in several plant species (see Introduction).

**Fig. 1.**
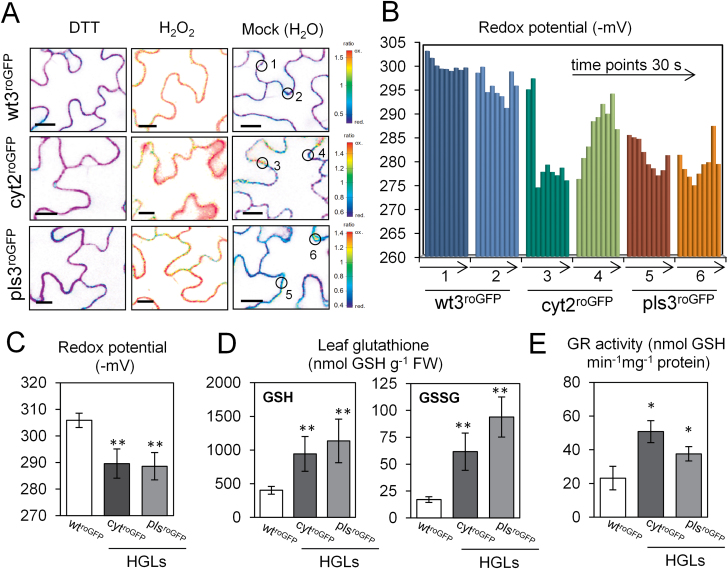
Analysis of cytosolic glutathione redox potential in epidermal cells of tobacco reveals a more oxidized state in HGLs compared with WT plants. (A) *In situ* measurements of glutathione redox potential, using cytosol-targeted GRX1-roGFP2 as sensor. The colour scale represents the fluorescence ratio with the redox state of roGFP ranging from fully reduced (red.) in blue to fully oxidized (ox.) in red. Calibration of the probe was performed with 100mM DTT and 50mM H_2_O_2_ for each sample. Bars, 10 µm. (B) Dynamic changes of glutathione redox potential for the points of analysis indicated by circles in (A). Each point was monitored for 270 s in 30 s time intervals. (C) Mean redox potentials of two independent lines of each pool (WT and HGL cyt and pls background with roGFP expression) calculated from ratio values for three individual plants of each line according to Meyer *et al.* (2007). (D) Mean contents of GSH and GSSG as determined by HPLC for plants as indicated in (C). (E) Glutathione reductase activities for WT and cyt and pls HGLs. Student’s *t*-test was used to calculate significant differences (*P<0.05, **P<0.01) between WT and HGLs. Error bar indicates standard deviation.

To assess the cytosolic redox state in epidermal leaf cells, WT tobacco and four different HGLs were stably transformed with the cytosol-targeted redox sensor GRX1-roGFP2. This method allows monitoring of the glutathione redox potential *in situ* at high spatial resolution via ratiometric analysis using confocal imaging (Meyer *et al.*, 2007; Schwarzlaender *et al.*, 2008). It was first confirmed that the dynamic range of the sensor as determined by DTT and H_2_O_2_ treatments was similar in WT and HGLs (Supplementary Fig. S1A at *JXB* online). The degree of oxidation of the GRX1-roGFP2 probe (Supplementary Fig. S1B) was used to calculate the glutathione redox potential. When monitored in the absence of stress exposure, oxidized cytoplasmic patches in HGLs were detected, which were not observed in WT (Fig. 1A). These patches displayed dynamic changes in their redox poise, with glutathione redox potentials varying in range from –275 to –295 mV (Fig. 1B). The mean glutathione redox potential in HGLs was less reducing compared with WT (–306 mV), reaching on average –290 mV for cyt^roGFP^ lines and –287 mV for pls^roGFP^ lines, respectively (Fig. 1C). Additionally, bulk analysis of glutathione from leaf extracts by HPLC revealed that the GSH:GSSG ratio was 20:1 in WT, while it dropped to 15:1 and 13:1 in the cyt^roGFP^ and pls^roGFP^ lines, respectively (Fig. 1D). Glutathione reductase activities were increased up to 2-fold in HGLs compared with WT (Fig. 1E).

### HGLs display increased levels of the activated MAPKs SIPK and WIPK

**Fig. 2.**
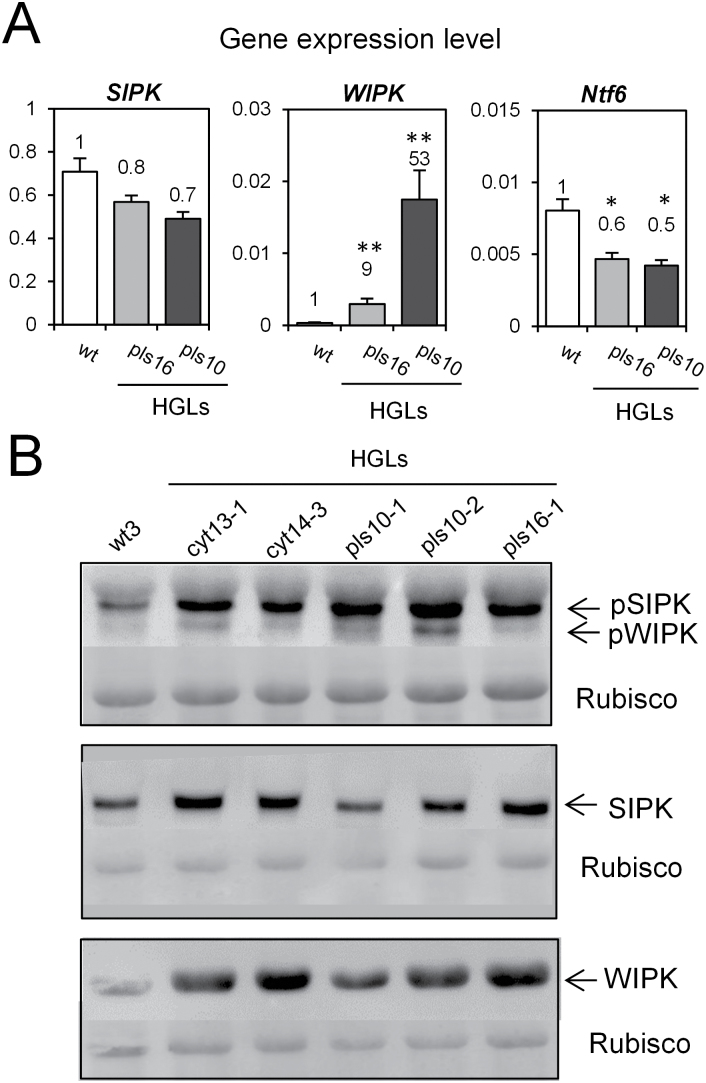
In leaves of tobacco HGLs, the MAPKs SIPK and WIPK are constitutively activated. (A) *WIPK*, *SIPK*, and *Ntf6* transcript abundance in HGLs and WT. Numbers indicate fold induction in comparison with normalized expression for WT. Ribosomal protein L25 was used as a reference gene. Results represent the mean value of three biological replicates±standard error. Constitutive expression of ribosomal protein L25 was confirmed by comparison with two additional reference genes, namely ubiquitin and elongation factor 1α (see Supplementary Fig. S2 at *JXB* online). (B) Immunoblot analysis of phosphorylated forms of MAPKs using anti-pTEpY-ERK1/2 antibody for the active kinases WIPK and SIPK in HGLs compared with WT (upper panel). SIPK and WIPK total protein levels were also determined (middle and lower panel). Forty micrograms of total protein for the active form of SIPK/WIPK and 15 µg of total protein for SIPK and WIPK detection were loaded per lane. For the loading control, the membrane was stained with amido black. Experiments were repeated three times with similar results, and results are presented for individual plants of each line. Student’s *t*-test was used to calculate significant differences (*P<0.05, ** *P*<0.01) between WT and HGLs.

To explore whether MAPK signalling is affected in HGLs, the expression of three tobacco MAPKs (SIPK, WIPK, and Ntf6) was first analysed at the transcript level (Fig. 2A). While expression of SIPK and Ntf6 kinase was barely affected in HGLs, *WIPK* transcripts were increased 9-fold and 53-fold in lines pls16 and pls10, respectively. Since *WIPK* gene induction correlates with and might be dependent on SIPK activation (Liu *et al.*, 2003), total protein amounts and activation states of both MAPKs were determined. When compared with WT, HGLs displayed increased levels of phosphorylated (i.e. active state) SIPK, and, albeit to a lesser extent, WIPK. Conversely, while accumulation of total WIPK protein was increased substantially in all HGLs, SIPK protein levels in WT and HGLs differed only slightly (Fig. 2B).

### In WT, glutathione application causes an oxidative shift in cytosolic redox potential and induces rapid activation of SIPK and WIPK

**Fig. 3.**
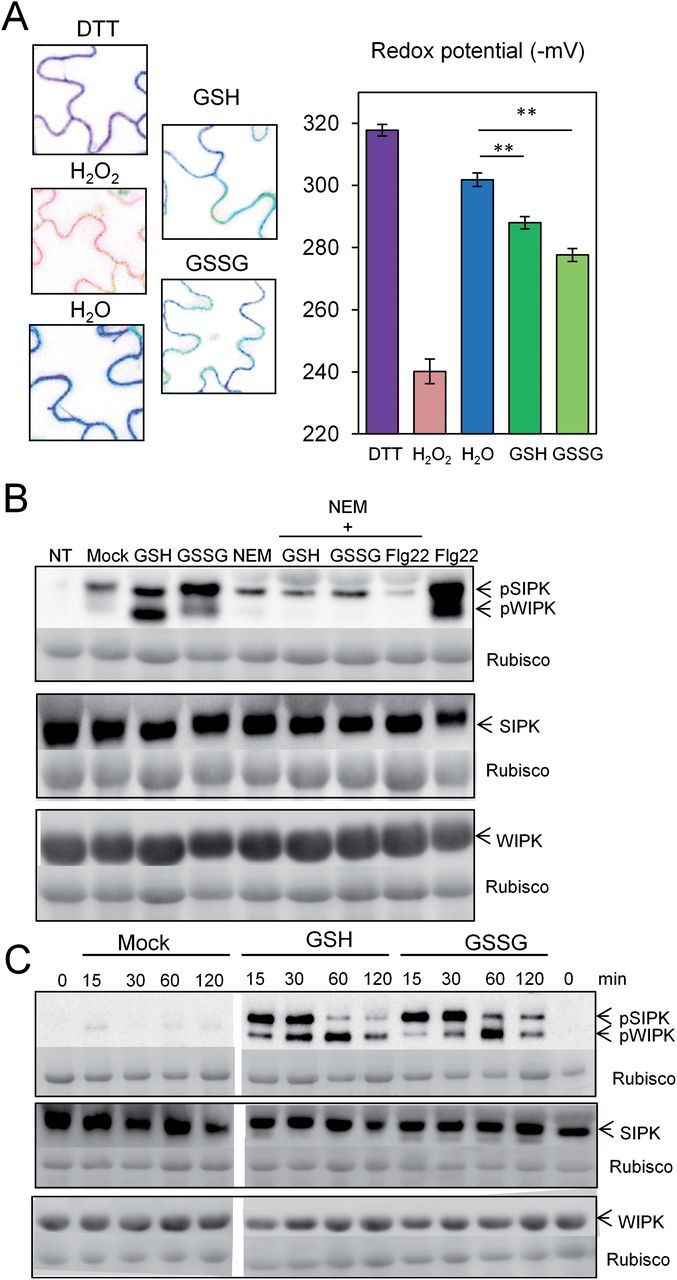
Infiltration of GSH (10mM) or GSSG (1mM) into leaves of WT tobacco causes significant oxidative shifts in cytosolic redox potential and rapidly activates SIPK and WIPK. (A) GSH and GSSG solutions were infiltrated with a needleless syringe from the abaxial leaf side. After 30min, redox potentials were determined with GRX1-roGFP2 as sensor in three independent samples (see also Fig. 1). Student’s *t*-test was used to calculate significant differences (**P<0.01) between treatments. (B) Treatment with GSH or GSSG for 30min activates MAPK signalling. Pre-incubation with 10mM *N*-ethylmaleimide (NEM) for 20min blocked SIPK and WIPK activation in response to GSH, GSSG, or 100nM Flg22 (flagellin N-terminal epitope). (C) Time course of SIPK and WIPK activation with GSH and GSSG over a 2h incubation period compared with mock treatment. Fifteen micrograms of total protein was loaded per lane. For loading control, the membrane was stained with amido black. Experiments were repeated twice with similar results.

To corroborate the assumption that the observed activation of SIPK and WIPK in HGLs is caused by the increase in cellular glutathione content and/or redox state, leaf discs of WT plants were treated with 10mM GSH or 1mM GSSG. Since GSH is not stable in aqueous solution (Yamamoto and Ishihara, 1994) and is expected to be further oxidized in the apoplast before uptake into the cytosol, treating leaf cells with GSH might, like GSSG, cause an oxidative shift of the cytosolic glutathione redox potential. To test this assumption, leaves of tobacco GRX1-roGFP2 transformants (in a WT background) were infiltrated with 10mM GSH or 1mM GSSG solution, and cytosolic glutathione redox potential was monitored over time. Indeed, both treatments caused shifts towards a more oxidized state (Fig. 3A).

A fast and strong activation of SIPK and WIPK was observed in response to reduced and oxidized glutathione, respectively, without a change in the total amounts of SIPK and WIPK proteins (Fig. 3B). Pre-treatment with NEM (preventing cysteine modifications, including disulfide bridge formation) blocked not only the activation of SIPK and WIPK by GSH, but also activation by GSSG. Likewise, NEM pre-treatment prevented the activation of both MAPKs by flagellin. A time course experiment revealed that the relative activation levels of SIPK and WIPK showed slightly different kinetics in response to GSH and GSSG. While, following GSSG treatment, SIPK activation was maintained somewhat longer compared with GSH treatment, the activation of WIPK appeared to be delayed (Fig. 3C).

### Non-infected HGLs display a constitutive upregulation of defence gene expression without an increase in free SA

**Fig. 4.**
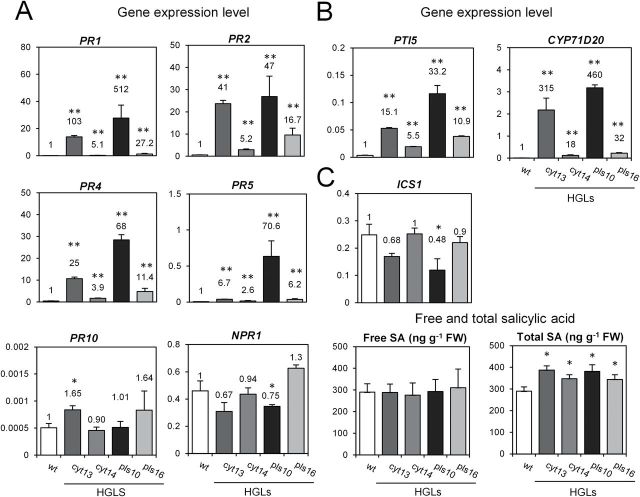
In tobacco HGLs, steady-state transcript levels for PR protein genes and immunity marker genes are upregulated without an increase in free SA. (A) Transcript levels for PR protein genes and *NPR1*. (B) Transcript levels for immunity marker genes *PTI5* and *CYP71D20.* (C) Transcript levels for *ICS1* and contents of free SA and total SA in WT and HGLs. For gene expression analysis, numbers indicate fold induction in comparison with normalized expression in WT. Ribosomal protein L25 was used as a reference gene. Results represent mean values of three biological replicates±standard error. For SA measurements, results represent mean values of five biological replicates±standard deviation. Student’s *t*-test was employed to calculate significant differences (*P<0.05, **P<0.01) between WT and HGLs. Experiments were repeated three to five times with similar results.

To assess the potential impact of MAPK signalling in HGLs as caused by the changed glutathione redox state on downstream defence signalling in the absence of pathogen challenge, an expression analysis was performed for selected genes considered to be specific for various defence-signalling pathways (van Loon, 1985). Expression of several PR protein genes was strongly (and constitutively) upregulated in HGLs when compared with WT (Fig. 4A). Upregulated genes included *PR1*, *PR2*, *PR4*, and *PR5*, known to be activated by SA in *NPR1*-dependent and -independent modes (Cao *et al.*, 1994; Rao *et al.*, 2002; Thibaud *et al.*, 2004). Two established PTI marker genes for *Nicotiana*, namely *CYP71D20* (Lacombe *et al.*, 2010) and *PTI5* (Nguyen *et al.*, 2010), were also significantly induced in HGLs (Fig. 4B), whereas transcripts for *PR10* and *NPR1* remained unchanged (Fig. 4A). Since SA-mediated signalling is thought to be required for induction of PR genes in response to biotrophic or hemi-biotrophic pathogens such as *P. syringae* (Katagiri *et al.*, 2002), the contents of free SA and total SA, including its glucoside form (SAG), were also determined. Interestingly, SA did not differ between HGLs and WT, consistent with unchanged (or even lowered) transcript amounts for isochorismate synthase (*ICS1*), an enzyme involved in SA biosynthesis. However, all HGLs had a significantly increased content of total SA, due to the presence of low amounts of the SA conjugate SAG, which was almost absent in WT (Fig. 4C).

### Upon pathogen challenge, HGLs differ from WT at the level of glutathione redox potential, PR gene expression, and MAPK activation

Based on the abovementioned observations, it was expected that redox change, coupled with MAPK activation and defence gene induction, would impact significantly on plant defence. To substantiate this assumption, the cytosolic redox state in epidermal cells of WT and HGLs (cyt and pls) was monitored during bacterial infection with *Psm* and *Pst*, respectively. In tobacco, the non-adapted pathovar *Psm* causes a typical HR, whereas the adapted pathovar *Pst* causes severe disease symptoms (for further details, see below).

**Fig. 5.**
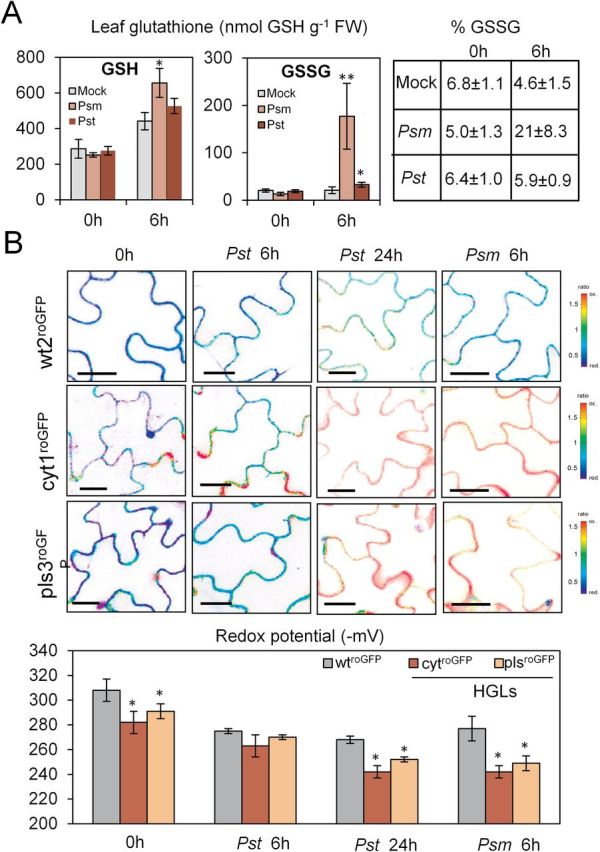
Response of cellular redox state of WT tobacco and HGLs to infection with adapted *Pst* or non-adapted *Psm* pathovars. (A) Accumulation of GSH and GSSG upon infection of WT with *Psm* and *Pst.* (B) GRX1-roGFP2-expressing plants (in WT or HGL background) were analysed immediately before (0h), 6h after *Psm* inoculation, and 6 and 24h after *Pst* inoculation. Mean redox potentials for the GSH/GSSG couple (mV) were calculated from three individual plants. For further details, see Fig. 1. Error bar indicates standard deviation. Bars, 10 µm. The pathogen inoculum was 5×10^7^ CFU ml^–1^. Student’s *t*-test was used to calculate significant differences (*P<0.05, **P<0.01) between WT and HGLs and different treatments.

In WT, infection with *Psm* (5×10^7^ CFU ml^–1^) caused a significant increase of glutathione, with GSSG content being increased 5-fold at 6h hpi. An increase in GSSG could also be detected after challenge with the adapted pathovar *Pst*; however, here the effect was less pronounced (Fig. 5A). At 6 hpi, the glutathione redox potential of WT increased from –308 mV to about –275 mV in response to both pathogens. HGLs, which already displayed a slightly oxidized state (–282/–291 mV) in the absence of pathogen challenge (see above), revealed further oxidation reaching –263/–270 mV for *Pst* infection and –242/–254 mV for *Psm* infection. At 24 hpi, challenge with *Pst* led to further oxidation in WT and HGLs, but cells remained viable (Fig. 5B). The early strong oxidation after infection with the non-adapted pathovar *Psm* correlated with an accelerated cell death, and no more roGFP fluorescence was detectable at 24 hpi due to the advanced stage of HR (Supplementary Fig. S3 at *JXB* online).

**Fig. 6.**
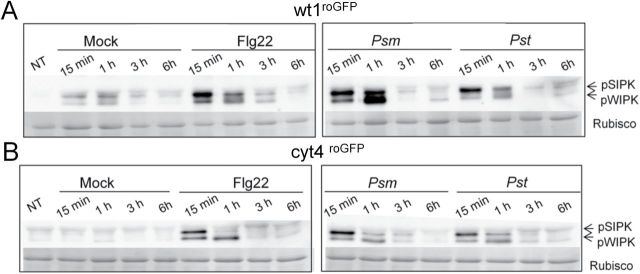
MAPK signalling in response to 100nM Flg22 treatment or pathogen challenge is more robust in WT than in HGLs. Time course of MAPK (SIPK and WIPK) activation in WT^roGFP^ (A) and cyt^roGFP^ HGL (B) after elicitation with 100nM Flg22, or after infection with *Psm* or *Pst*. Fifteen micrograms of total protein was loaded per lane for detection of activated SIPK and WIPK. For the loading control, the membrane was stained with amido black.

In contrast to the constitutive activation of SIPK and WIPK in HGLs (Fig. 2), the further amplification of MAPK signalling upon challenge with flagellin, *Psm*, or *Pst* did not exceed the response of WT. After infection with the non-adapted pathovar *Psm*, HGLs even displayed an attenuated response (Fig. 6). While, in WT, activation of MAPK signalling was particularly strong with this pathovar, the adapted pathovar *Pst* induced less activation of both kinases, probably due to effector suppression via AvrPto (Xiang *et al.*, 2008).

**Fig. 7.**
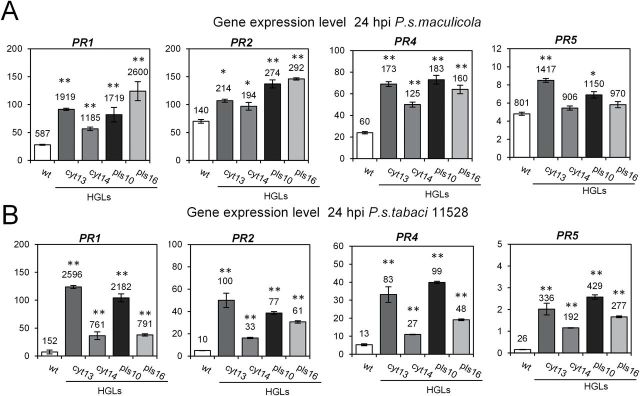
Upon pathogen challenge, PR protein genes show stronger expression in HGLs compared with WT. (A) Strong upregulation of four PR protein-coding genes at 24 hpi with non-adapted pathovar *Psm*. (B) Upregulation of the same PR protein-coding genes after infection with the adapted pathovar *Pst.* Bacterial titre infiltrated into the leaves was 5×10^5^ CFU ml^–1^. Numbers indicated gene induction relative to expression in WT in the absence of pathogen challenge. Ribosomal protein L25 was used as the reference gene. Results represent mean values of three biological replicates±standard error. Experiments were repeated two times with similar results.

To explore whether HGLs respond to infection with a further increase in defence gene expression, transcript amounts for PR protein genes were determined at 24 hpi with *Pst* (strain 11528) or *Psm*. HGLs displayed a substantial further induction of PR protein genes in response to both pathovars, the effect being even more pronounced for infection with *Psm* (Fig. 7A; for comparison with non-infected plants, see Fig. 4). Likewise, for WT, the induction of PR protein gene expression was stronger after challenge with the non-adapted pathovar (*Psm*) compared with the adapted pathovar *Pst* (Fig. 7B).

### HGLs are less susceptible to adapted and non-adapted pathovars of *P. syringae*

To explore whether the changed glutathione redox potential in HGLs was of physiological relevance for subsequent defence reactions, the response of transgenic lines to pathogen challenge was analysed at different levels. To monitor defence reactions, an infection procedure via leaf infiltration was employed (inoculum: 10^8^–10^3^ CFU ml^–1^). Disease symptoms were compared for two adapted *Pst* strains, and the non-adapted pathovars *Psm* and *Pss*. In WT tobacco, *Pst* causes tobacco wildfire disease, strain *Pst* ATCC 11528 being highly virulent (Thilmony *et al.*, 1995), whereas *Pst* ATCC 11527 causes only slowly developing disease symptoms. Conversely, the non-adapted pathovars *Psm* and *Pss* are fought off by the tobacco host via a typical HR (Oh *et al.*, 2006).

**Fig. 8.**
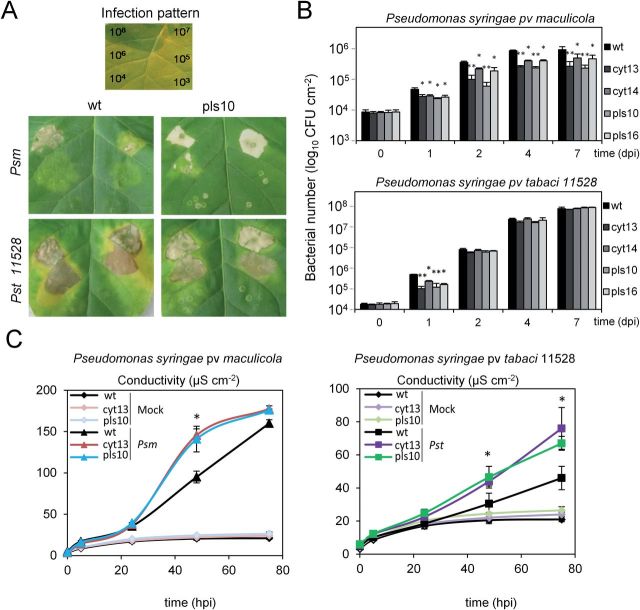
Visible symptoms, time course of bacterial propagation, and HR response in WT and HGLs inoculated with the non-adapted pathovar *Psm* and the adapted pathovar *Pst.* (A) Infection symptoms after 7 d in WT tobacco and HGL pls10 in response to different inoculum of *P. syringae* pathovars (see top panel for infection pattern). Inoculum varied from 10^8^ to 10^3^ CFU ml^–1^ (for more HGLs, see Supplementary Figs S4 and S5 at *JXB* online).). (B) Time course of bacterial propagation in infected leaf areas, inoculated with *Psm* (5×10^5^ CFU ml^–1^, 50 µl) or *Pst* 11528 (10^4^ CFU ml^–1^, 50 µl). Results represent mean values of four biological replicates ± standard deviation. (C) Time course of electrolyte leakage, marker for cell death in HR, monitored as increase in conductivity after infection with 5x 10^5^ CFU ml^–1^ of the non-adapted *Psm* or the adapted pathovar *Pst*. Results represent mean values of three biological replicates ± standard deviation. Student’s *t*-test was employed to calculate significant difference between WT and HGLs (significant differences marked with asterisks * *P*<0.05, ** *P*<0.01). Experiments were repeated twice with similar results.

In WT plants, *Pst* ATCC 11528 at high titre caused necrosis of the entire infiltrated leaf area, surrounded by spreading chlorosis. In marked contrast, necrotic lesions appeared brighter in HGLs, resembling a HR, and chlorotic areas were spatially more restricted than in WT (Fig. 8A and Supplementary Fig. S4 at *JXB* online). This deviation from WT was most pronounced in HGL pls10, which displayed the highest accumulation of glutathione (Supplementary Fig. S6A at *JXB* online). For HGLs, bacterial growth was only transiently reduced when compared with WT: for strain *Pst* 11528 only at day 1 post-infection (Fig. 8B, lower pannel) and for the less virulent strain *Pst* 11527 until day 2 post-infection (Supplementary Fig. S5, upper panel).

Upon infection with the non-adapted pathovars *Psm* and *Pss* (inoculum: 10^8^ and 10^7^ CFU ml^–1^, respectively), WT and HGLs all developed lesions of the HR (Fig. 8A and Supplementary Fig. S4); however, HR symptoms were more pronounced in HGLs pls10 and cyt13. Remarkably, bacterial growth remained significantly reduced in all HGLs over the entire 7 d infection period (Fig. 8B, upper panel, and Supplementary Fig. S5, lower panel), indicating a sustained response of HGLs towards bacterial challenge.

Visual inspection of symptom development after infection with *Psm* revealed that HR lesions occurred faster and were more prominent in HGLs compared with WT. Since HR causes cell death in tobacco (Mysore and Ryu, 2004), electrolyte leakage was monitored as a cell death marker. In HGLs infected with *Psm*, electrolyte leakage increased significantly at 48 hpi (Fig. 8C, left panel). Remarkably, HGLs also displayed increased electrolyte leakage upon infection with the adapted pathovar *Pst*; however, this increase was less pronounced. At 72 hpi, electrolyte leakage was 67 and 45% higher for cyt13 and pls10, respectively, compared with WT (Fig. 8C, right panel).

### HGLs are primed for SA accumulation and callose deposition

**Fig. 9.**
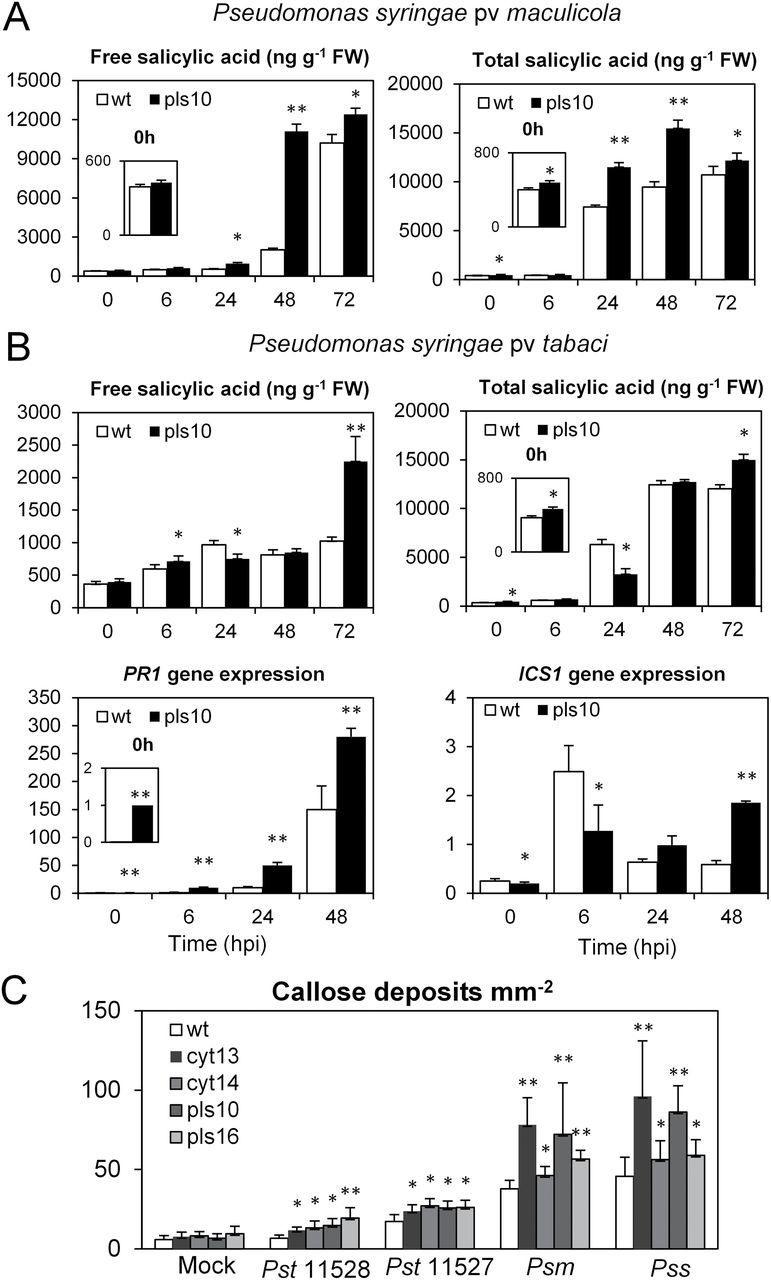
Upon pathogen challenge, HGLs display increased SA accumulation and callose deposition compared with WT. (A) Increase in accumulation of free and total SA after challenge with the non-adapted pathovar *Psm* in WT compared with HGL pls10. (B) After challenge with the adapted pathovar *Pst*, HGL pls10 accumulates increased amounts of SA at 72 hpi, temporally correlating with HR. Time courses for *ICS1* (bottom panel right) and *PR1* expression (bottom panel left) are included. For SA and SAG analysis, results represent mean values of three independent samples±standard deviation. Student’s *t*-test was employed to calculate significant difference (*P<0.05) between WT and HGL pls10. For gene expression analysis, numbers indicate fold induction in comparison with normalized expression in WT. Ribosomal protein L25 was used as the reference gene. Results represent mean values of three biological replicates±standard error. Experiments were repeated two times with similar results. (C) Callose deposits per mm^2^ in different HGLs and WT after infection with different *P. syringae* pathovars. The initial inoculum was 5×10^5^ CFU ml^–1^; for mock treatment, 10mM MgCl_2_ was applied. Results represent mean values of 10 independent samples for each line±standard deviation. *P<0.05 and **P<0.01 indicate the level of significance calculated with Student’s *t*-test.

After challenge with the non-adapted *P. syringae* pathovar, HGLs accumulated more SA and SAG than WT (Fig. 9A), correlating with the stronger HR reaction (Fig. 8A). To dissect the infection process for the adapted pathovar *Pst* in more detail, accumulation of SA and SAG and expression of *ICS1* were monitored (Fig. 9B). In WT, *ICS1* expression was strongly upregulated as early as 6 hpi but declined thereafter and remained at a lower level for the following 48h. This time course correlated with an increase in SA only during the first 24h, with no further change until 72 hpi. In marked contrast, in HGL pls10, the initial peak of *ICS1* expression at 6 hpi was followed by a second activation at 48 hpi (Fig. 9B), this second activation correlating with a further increase in free SA at 72 hpi. Interestingly, this second phase of SA accumulation coincided temporally with the development of the HR-like symptoms (Fig. 8A) and electrolyte leakage (Fig. 8C). Note that, in comparison with WT, *PR1* expression was higher in HGL pls10 during the entire course of infection (Fig. 9B): at 6 hpi, *PR1* transcript amount in line pls10 was 2600-fold higher than in WT.

The observed attenuation of disease symptoms in HGLs suggested that the high glutathione levels (Supplementary Fig. S6B) might also affect callose formation, thereby preventing bacterial spread. Indeed, at 24 hpi, increased callose deposition was observed in HGLs in response to challenge with adapted and non-adapted *P. syringae* pathovars (Fig. 9C). Particularly high levels of callose deposits were observed when plants were infected with the non-adapted pathovars *Psm* and *Pss*. Remarkably, the number of callose deposits in HGLs cyt13 and pls10 was almost doubled compared with WT. The lowest number of callose deposits was detected when plants were infected with the adapted pathovar *Pst* strain ATCC 11528, whereas during infection with *Pst* ATCC 11527, a less virulent strain, callose formation was slightly more induced than with ATCC 11528 (Fig. 9C). Note that, in the absence of infection, mock treatment did not cause callose deposition in HGLs, indicating that transgenic lines were primed for callose deposition.

Considering the importance of ROS accumulation and activation in immunity (Torres, 2010), we measured H_2_O_2_ accumulation before and after pathogen challenge in WT and HGLs. Remarkably, detection of H_2_O_2_ before and after infection did not reveal significant differences between HGLs and WT, except for the transient increase at 1 hpi, when H_2_O_2_ levels were slightly lower in HGLs compared with WT (Supplementary Fig. S7 at *JXB* online), possibly due to rapid quenching by glutathione.

## Discussion

This study demonstrated that constitutively upregulated glutathione content as implemented in transgenic tobacco HGLs as well as short-term treatment of WT tobacco with glutathione caused an oxidative shift of the cytosolic glutathione redox potential and resulted in activated MAPK signalling via SIPK and WIPK. This activation indicates a possible redox-regulated mechanism. In HGLs, glutathione-activated MAPK signalling in the absence of pathogens was not accompanied by a change in free SA content but caused an increased expression of several PR genes. Furthermore, the observed oxidative shift in HGLs, combined with high glutathione levels, directly or indirectly improved plant defence upon challenge with adapted or non-adapted *P. syringae* pathovars. This upgrading of defence responses included further amplification of defence protein expression, callose deposition, SA accumulation, and a HR.

### Tobacco HGLs, a tool for exploring the impact of glutathione-based redox signalling on immune response

Several strategies have aimed to increase cellular glutathione content in plants (Noctor *et al.*, 1998; Creissen *et al.*, 1999; Herschbach *et al.*, 2010) in order to explore the impact of high glutathione on plant performance under stress. The introduction of StGCL-GS-expressing tobacco HGLs (Liedschulte *et al.*, 2010) has offered a novel approach to address the effects of increased glutathione accumulation on defence operations in the absence of sulfur-based secondary metabolism (important in the Brassicaceae; see Introduction). Due to expression of the bifunctional StGCL-GS enzyme, glutathione biosynthesis is uncoupled from redox-mediated post-translational control (Liedschulte *et al.*, 2010) and feedback inhibition by glutathione, regulatory processes that—in the absence of stress exposure—keep the endogenous GCL enzyme under tight control (Gromes *et al.*, 2008). This imposed uncoupling of glutathione biosynthesis from its endogenous controls allows accumulation of cellular glutathione to substantially increased levels (Fig. 1 and Supplementary Fig. S6), levels normally observed only upon stress exposure, e.g. oxidative stress caused by ozone, paraquat treatment, or pathogen attack (Vanacker *et al.*, 2000; Bick *et al.*, 2001).

### Cellular redox homeostasis in HGLs: evidence for an oxidative shift in the cytosol

Monitoring the glutathione redox potential of leaf epidermal cells from HGLs with the GRX1-roGFP2 cytosolic sensor (Meyer *et al.*, 2007; Schwarzlaender *et al.*, 2008) revealed that, in this cell type, the average glutathione redox potential was less negative compared with control lines. In addition, subcellular patches with an even higher oxidation state were repeatedly observed, pointing to cytosolic microenvironments of different redox potential. The results from quantitative bulk analysis of GSH and GSSG support a pronounced shift towards GSSG in the entire leaf (Fig. 1). Recently, Noctor *et al.* (2013) suggested that the disulfide form of glutathione may largely be sequestrated in the vacuole. The results presented here indicate that dynamic changes in the cytosolic GSH/GSSG ratio do also occur. While increased GR activities in HGLs reflect a cellular response to the oxidative shift, it remains to be shown whether cytosolic availability of NADPH may have been limiting. The observed redox shift may result from metabolic perturbations, e.g. interference with photorespiration induced by a glutathione synthesis-related decrease in cytosolic glycine levels (Noctor *et al.*, 1998). Interestingly, in HGLs, the expression of the peroxisomal catalase isoform *CAT1*, corresponding to the *CAT2* gene in *Arabidopsis* (Willekens *et al.*, 1995), is 2-fold increased at the transcript level (data not shown).

While in tobacco HGLs a slightly oxidized cytosol correlated with an improved defence against adapted and non-adapted pathovars of *P. syringae*, an increase in glutathione redox potential in the glutathione-deficient *pad2-1* mutant has been correlated with an impaired defence against pathogens (Dubreuil-Maurizi *et al.*, 2011). This discrepancy may indicate that, in response to pathogen attack, both a shift towards oxidation and an increased glutathione content are required to be realized in tobacco HGLs, whereas in the *pad2-1* mutant the low content of glutathione may have compromised cellular defence reactions.

Remarkably, comparable oxidative shifts in cytosolic glutathione redox potential were also observed after short-term treatment of WT tobacco leaf discs with either GSH or GSSG (Fig. 3). These transient shifts in cytosolic glutathione redox potential were of a similar magnitude compared with those observed in HGLs in the absence of pathogen challenge (Fig. 1). This may be explained by rapid oxidation of GSH in aqueous solution (Yamamoto and Ishihara, 1994) and bypassing the oxidizing environment of the apoplast before entering the cells.

### In HGLs, the oxidative shift in the cytosol combined with elevated glutathione levels activates MAPK signalling

In the absence of pathogen attack, HGLs displayed an increased activation of SIPK and to a lesser extent of WIPK, accompanied by a significant increase in WIPK transcript and protein (Fig. 2). These constitutive changes in MAPK signalling resembled those observed in WT upon short-term treatment with GSH or GSSG (Fig. 3). The rapid activation of SIPK and WIPK in WT after glutathione treatment indicated redox-mediated regulation. Thus, the oxidative shift in cytosolic redox potential may have caused sulfenylation of cysteine residues in enzymes involved in MAPK signalling that can be glutathionylated with GSH or GSSG (Grek *et al.*, 2013), as in WT leaf discs NEM pre-treatment almost completely prevented activation of both MAPKs. Whether the mechanism involved direct redox control of MAPKs, activation of upstream kinases, and/or inactivation of MAPK phosphatases remains to be shown. A recent study on the tobacco MAPK phosphatase NtMKP1 revealed that its suppression resulted in enhanced activation of WIPK and SIPK after wounding (Oka *et al.*, 2013). While for mammals compelling evidence has been provided for redox-mediated inactivation of a MAPK phosphatase, involving a glutathionylation mechanism (Kim *et al.*, 2012), activation of upstream kinases (MAPKK/K) and inactivation of MAPK phosphatases are not necessarily mutually exclusive. Heart p38α-MAPK, a serine-threonine kinase that is activated by its upstream activator MKK3, responds to multiple stresses including oxidants. In a recent study, it was shown that activation of p38α-MAPK is dependent on redox-sensing cysteines within p38α, namely Cys119 and Cys162, both close to the known MKK3 docking domain, which act as electron donors and form a disulfide bridge with MKK3 (Bassi *et al.*, 2014).

### Constitutive expression of PR genes in HGLs supports an SA-independent induction route

In the absence of pathogen challenge, HGLs displayed a substantial increase in the expression of several PR genes and two established markers for PTI in the absence of any change in free SA (Fig. 4). A plausible explanation for the lack of SA increase in HGLs in the absence of infection would be suppression of its accumulation by constitutively active SIPK. A negative impact of WIPK and SIPK on SA accumulation in tobacco has been suggested previously due to an increase in SA levels after silencing of those MAPKs (Seo *et al.*, 2007).

In the absence of pathogen challenge, expression of *NPR1* was barely affected in HGLs. Recently, Tsuda *et al.* (2013) reported that, in *Arabidopsis*, constitutive MPKK-mediated activation of MAPK3/6 was sufficient to activate most of the SA-responsive genes in a SA-independent manner. As this activation also occurred in the *npr1* mutant, induction of defence genes via the MAPK cascade and the NPR1-mediated induction of SA-responsive genes are thought to represent separate signalling routes. The results obtained for tobacco corroborate this observation, in agreement with a SIPK-dependent/SA-independent activation of PR gene expression in the absence of infection.

Since the evidence for two separate routes (MAPK-dependent and SA-dependent) leading to activation of defence genes in HGLs is compelling, it can be assumed that changes in cellular glutathione content or glutathione redox potential will affect both signalling routes via different molecular mechanisms. Activation of NPR1 target genes is known to require NPR1 monomerization prior to entry into the nucleus, phosphorylation at the N-terminal phosphodegron, and subsequent proteasome-mediated turnover (Spoel *et al.*, 2009). More recently, Lindermayr *et al.* (2010) reported that, in *Arabidopsis*, NPR1 and TGA1, both redox-controlled regulators of systemic acquired resistance, are subject to *S*-nitrosylation and *S*-glutathionylation at *S*-nitrosoglutathione concentrations in the low micromolar range. Also, it has been shown that conditional activation of SIPK may generate nitric oxide (Asai *et al.*, 2008), and that NPR1 translocation to the nucleus is promoted by nitric oxide (Lindermayr *et al.*, 2010). Thus, it may be speculated that, upon pathogen challenge, activation of MAPK(s) generates nitric oxide, which in the presence of a substantially increased GSH pool leads to *S*-nitrosoglutathione formation.

### In HGLs, improved defence against adapted and non-adapted *P. syringae* pathovars correlates with amplified oxidative shift and SA accumulation

Positive correlation of increased levels of glutathione and SA has been related to abiotic stress tolerance in several Brassicaceae species (Freeman *et al.*, 2005). Additionally, constitutive SA accumulation in the *cpr* and *dnd1* mutants affected the glutathione pool size (Mateo *et al.*, 2006). Recently, Han *et al.* (2013) demonstrated that when oxidative stress-induced glutathione accumulation is prevented in a *cat2 cad2* double mutant, this resulted in decreased SA content and *ICS1* transcript amounts, whereas the single *cat2* mutant accumulated substantial levels of SA. Thus, while pronounced oxidative stress causes both induction of glutathione biosynthesis and accumulation of SA, stress-induced formation of glutathione appears to be required for SA accumulation.

Upon challenge with adapted and non-adapted *P. syringae* pathovars, HGLs mobilized their defences faster and stronger than WT plants. This was accompanied by augmented transcript increases for SA-dependent genes (Fig. 7), expeditious SA accumulation, and more callose depositions (Fig. 9), suggesting a strong SA-dependent defence response upon infection. On the other hand, the strong but transient activation of MAPK signalling observed in WT tobacco upon infection appeared to be attenuated in HGLs (Fig. 6).

**Fig. 10.**
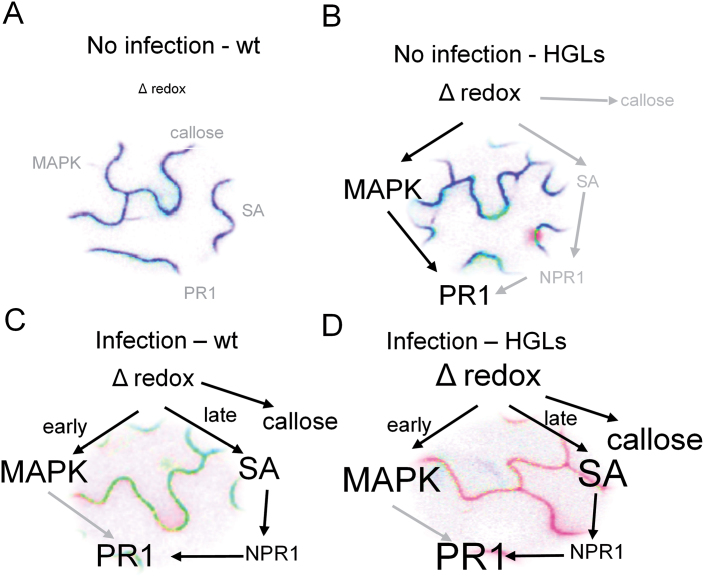
Impact of imposed glutathione accumulation on redox homeostasis in the cytosol and *P. syringae*-related defence events: a model. In the absence of biotic stress, MAPK and SA signalling pathways are not induced (grey) in the WT plants (A), whereas in HGLs, glutathione accumulation causes an oxidative redox shift in the cytosol, correlating with MAPK activation and defence gene induction (black) without elevation in free SA (B). Upon infection, transient MAPK activation (early response) precedes SA accumulation and defence gene accumulation in WT (late response) (C). This response is amplified in HGLs, causing stronger oxidation of the cytosol compared with WT, and correlating with more prominent accumulation of free SA and PR gene expression (D).

In summary, the results indicate that glutathione accumulation *per se* does not necessarily induce SA accumulation, but that it does so in the presence of pronounced oxidative stress as occurring during pathogen attack. In which way the constitutive MAPK signalling and PR gene expression in HGLs in the absence of pathogens contribute to the improved defence upon pathogen attack remains to be shown. Apparently, a slightly oxidized cytosol, together with an increased total glutathione pool size, benefits faster activation of defence upon infection as shown in a proposed model (Fig. 10). This is in accordance with the recent report of Pastor *et al.* (2013) who suggested that an augmented defence response after treatment with priming inducer BABA in *Arabidopsis thaliana* might be associated with an oxidized state. It appears that in our system the primed state has been achieved simply by imposed glutathione production without the involvement of ROS, offering a redox environment suitable for faster activation of downstream reactions in defence.

## Supplementary data

Supplementary data are available at *JXB* online.

Supplementary Fig. S1. Ratiometric analysis of WT and HGLs using GRX1-roGFP2 as sensor.

Supplementary Fig. S2. Steady-state transcript levels for additional reference genes ubiquitin and elongation factor 1α.

Supplementary Fig. S3. Raw data fluorescence images of roGFP2 in the cytosol of WT plants after different infiltration treatments.

Supplementary Fig. S4. Disease symptoms in tobacco leaves 7 d after infection with two strains of the adapted pathovar *Pst* strains ATCC 11528 and 11527 and two non-adapted pathovars (*Psm* and *Pss*) in WT compared with all four HGLs under study.

Supplementary Fig. S5. Time course of bacterial propagation in infected areas, inoculated with *Pst* strain 11527 and *Pss*.

Supplementary Fig. S6. Glutathione content in tobacco WT and transgenic HGLs.

Supplementary Fig. S7. Hydrogen peroxide detection after infection with the non-adapted pathovar *Psm*.

Supplementary Table S1. List of primers used in the experiments.

Supplementary Data

## Abbreviations:

CFU: colony-forming units
DCFH-DA: dichlorodihydrofluorescein diacetate
DTT: dithiothreitol
GSH: reduced glutathione
GSSG: oxidized glutathione
HGL: high-glutathione line
hpi: h post-infection
HPLC: high-performance liquid chromatography
HR: hypersensitive response
MAPK: mitogen-activated protein kinase
NEM: N-ethylmaleimide
P/MAMP,: pathogen/microbe-associated molecular patterns
PR: pathogenesis-related
Psm: *Pseudomonas syringae* pv. *maculicola*
Pss: *Pseudomonas syringae* pv. *syringae*
Pst: *Pseudomonas syringae* pv. *tabaci*
PTI: pattern-triggered immunity
ROS: reactive oxygen species
SA: salicylic acid
SAG: salicylic acid glucoside
SIPK: SA-induced protein kinase
WIPK: wound-induced protein kinase
WT: wild type.
