# Supplementary material for: Imposed glutathione-mediated redox switch modulates the tobacco wound-induced protein kinase and salicylic acid-induced protein kinase activation state and impacts on defence against Pseudomonas syringae
Source: J Exp Bot. 2015 Jan 26;66(7):1935–50. doi: 10.1093/jxb/eru546 (PMC4378631; doi:10.1093/jxb/eru546)
Supplement: Supplementary Data [file supp_66_7_1935__index.html]

Imposed glutathione-mediated redox switch modulates the tobacco wound-induced protein kinase and salicylic acid-induced protein kinase activation state and impacts on defence against Pseudomonas syringae — Imposed glutathione-mediated redox switch modulates the tobacco wound-induced protein kinase and salicylic acid-induced protein kinase activation state and impacts on defence against Pseudomonas syringae — Supplementary Data 

# Imposed glutathione-mediated redox switch modulates the tobacco wound-induced protein kinase and salicylic acid-induced protein kinase activation state and impacts on defence against *Pseudomonas syringae*

## Supplementary Data

Data files

**Files in this Data Supplement:**

- Supplementary Data - Supplementary Data
