# Supplementary material for: Imposed glutathione-mediated redox switch modulates the tobacco wound-induced protein kinase and salicylic acid-induced protein kinase activation state and impacts on defence against Pseudomonas syringae
Source: J Exp Bot. 2015 Jan 26;66(7):1935–50. doi: 10.1093/jxb/eru546 (PMC4378631; doi:10.1093/jxb/eru546)
Supplement: Supplementary Data [file supp_eru546_jexbot139386_file001.pdf]

SUPPLEMENTARY DATA

Imposed glutathione-mediated redox switch modulates tobacco SIPK/WIPK activation state and impacts on defense against *Pseudomonas syringae*

Sanja Matern[1,2], Tatjana Peskan-Berghoefer[1], Roland Gromes[1], Rebecca Vazquez Kiesel[1] and Thomas Rausch#[1]

[1]Centre for Organismal Studies Heidelberg, Department of Plant Molecular Physiology, University of Heidelberg, 69120 Heidelberg, Germany

[2]The Hartmut Hoffmann-Berling International Graduate School of Molecular and Cellular Biology (HBIGS), University of Heidelberg, 69120 Heidelberg, Germany

Supplementary Fig. S1

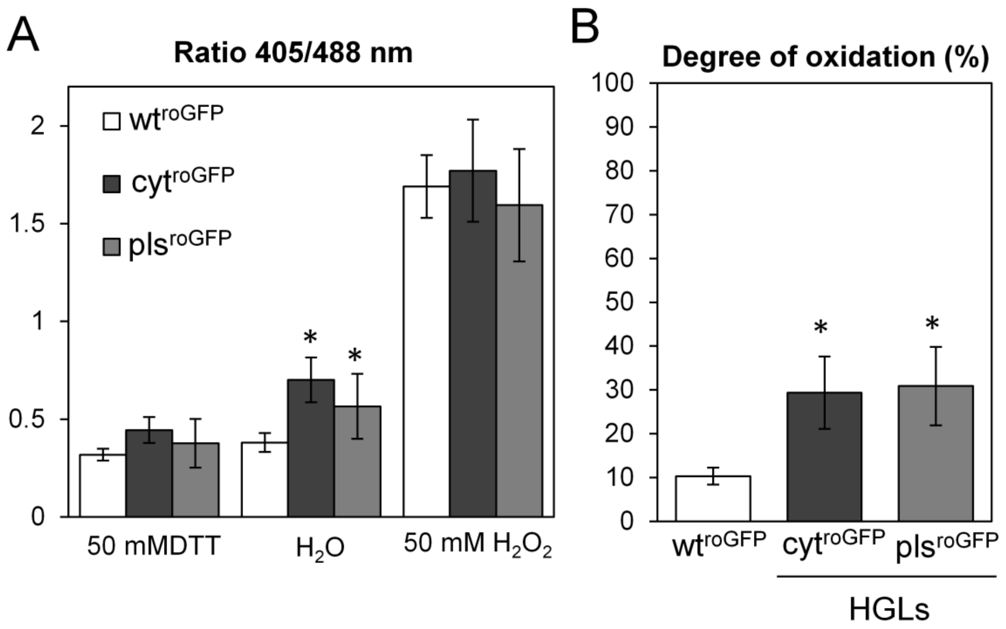

Ratiometric analysis of WT and HGLs using GRX1-roGFP2 as sensor

(A) Dynamic range of ratio 405/488 nm of roGFP probe as determined by calibration with 100 mM DTT and 50 mM H_2O_2, including mock treatment with water (H_2O). Fluorescent images of roGFP2 were collected for the cytoplasm and analyzed with custom Matlab analysis suite as described by Schwarzlaender et al. (2008).

(B) Degree of oxidation of GRX1-roGFP2 probe calculated from ratio values in (A) and used for the calculation of the redox potential according to Meyer et al. (2007).

Results represent mean values of three plant individuals of each line, with two independent samples each. Student t-test was used to calculate significant difference ($p<0.05$) between WT and HGLs (indicated with asterisk).

Supplementary Fig. S2

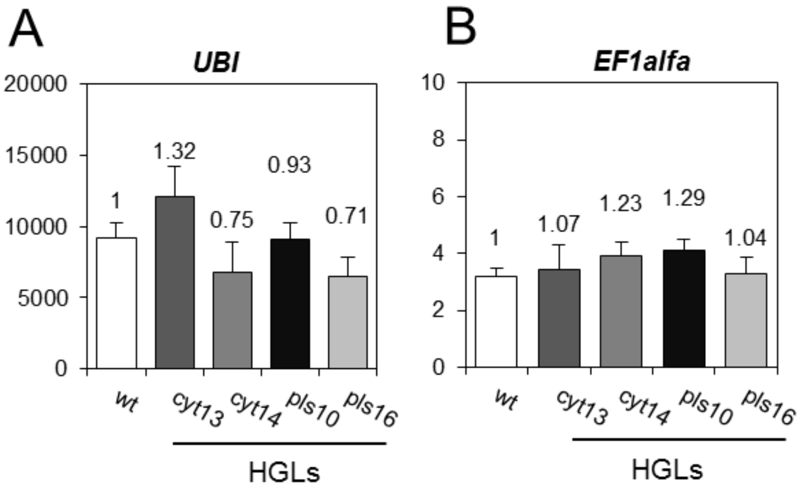

Steady-state transcript levels for additional reference genes ubiquitin and elongation factor 1α

For gene expression analysis, numbers indicate fold-induction in comparison with normalized expression in WT. Ribosomal protein L25 was used as a reference gene. Results represent mean values of three biological replicates ± standard error.

Supplementary Fig. S3

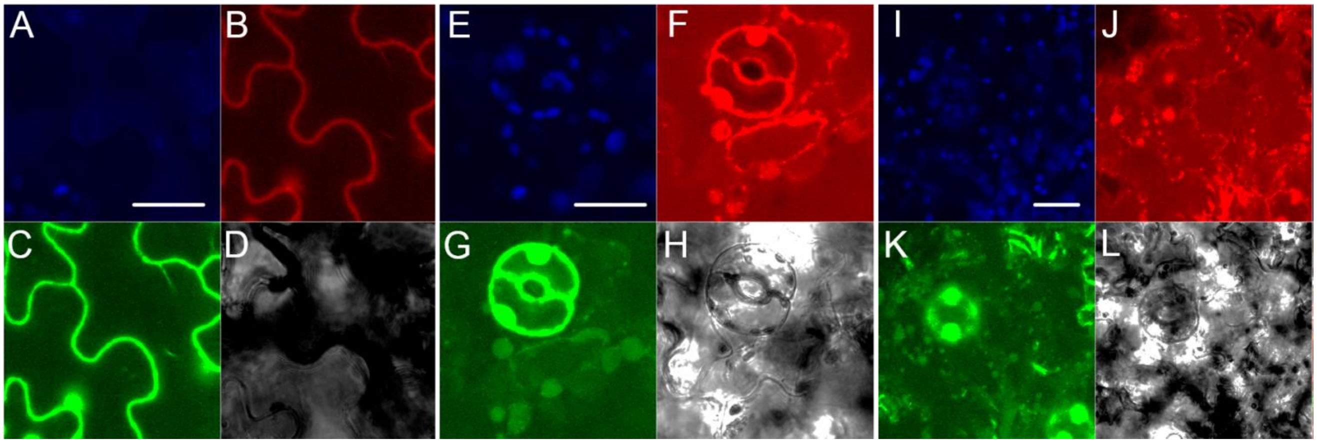

Raw data fluorescence images of roGFP2 in the cytosol of WT plants after different infiltration treatments

(A – D) roGFP2 fluorescence in WT background plants under control conditions (before infection).

(E – L) roGFP2 fluorescence in WT background plants 24 hpi with *Psm* (note loss of fluorencence in the epidermal cells, while stomata are functional and widely open).

In blue (A, E, I) images of autofluorescence taken with excitation wavelength 405 nm and emission wavelength of 435-485 nm.

In red (B, F, J) images of roGFP fluorescence taken with with excitation wavelength 405 nm and emission wavelength of 500-530 nm.

In green (C, G, K) images of roGFP fluorescence taken with with excitation wavelength 488 nm and emission wavelength of 500-530 nm.

In D,H and L merged images. Scale bars indicate 10 µm.

Supplementary Fig. S4

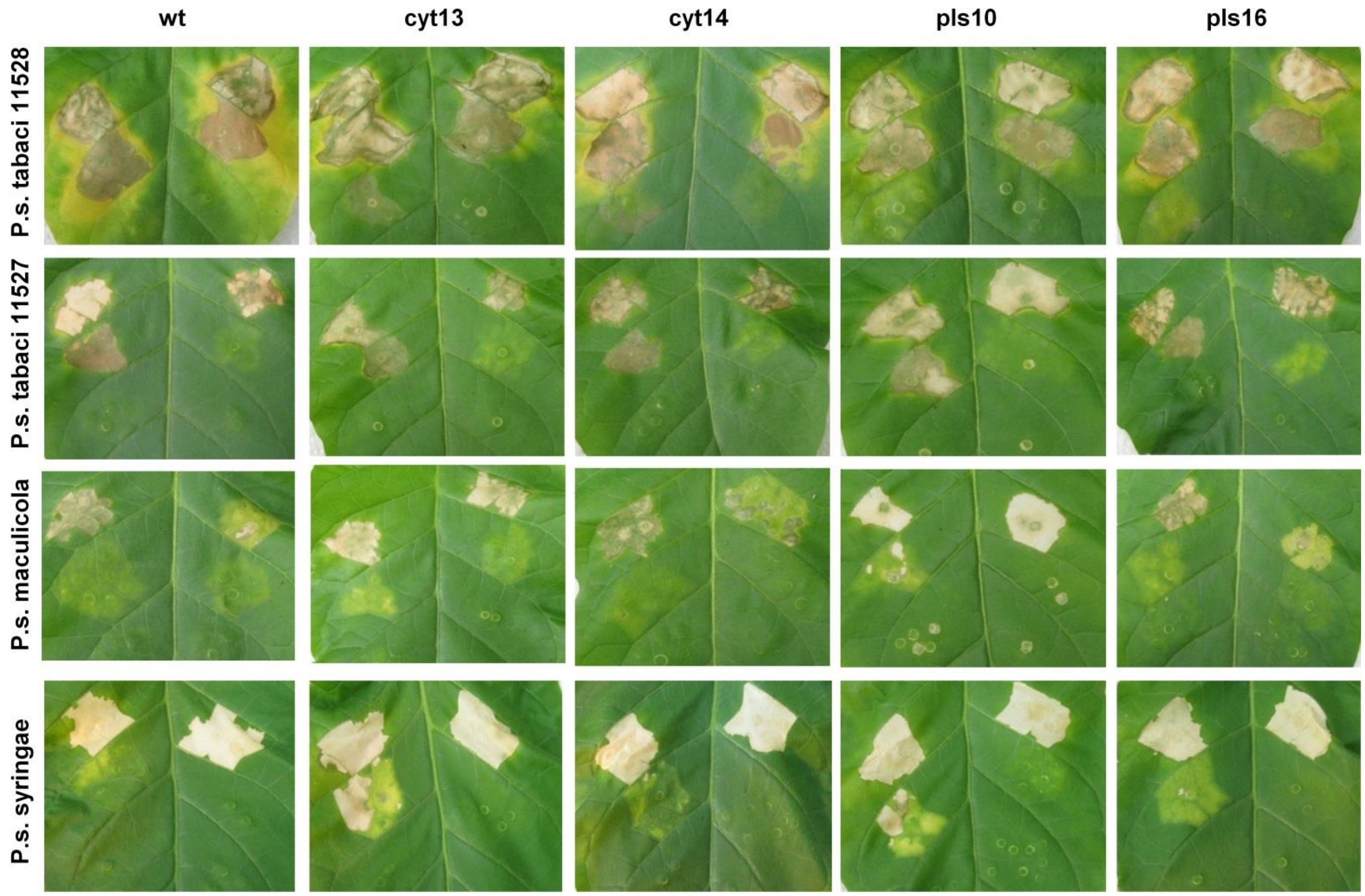

Disease symptoms in tobacco leaves seven days after infection with two strains of the adapted pathovar *Pst* and two non-adapted pathovars (*Psm* and *Pss*) in WT as compared to all four HGLs under study

For further details see Fig. 8A.

Supplementary Fig. S5

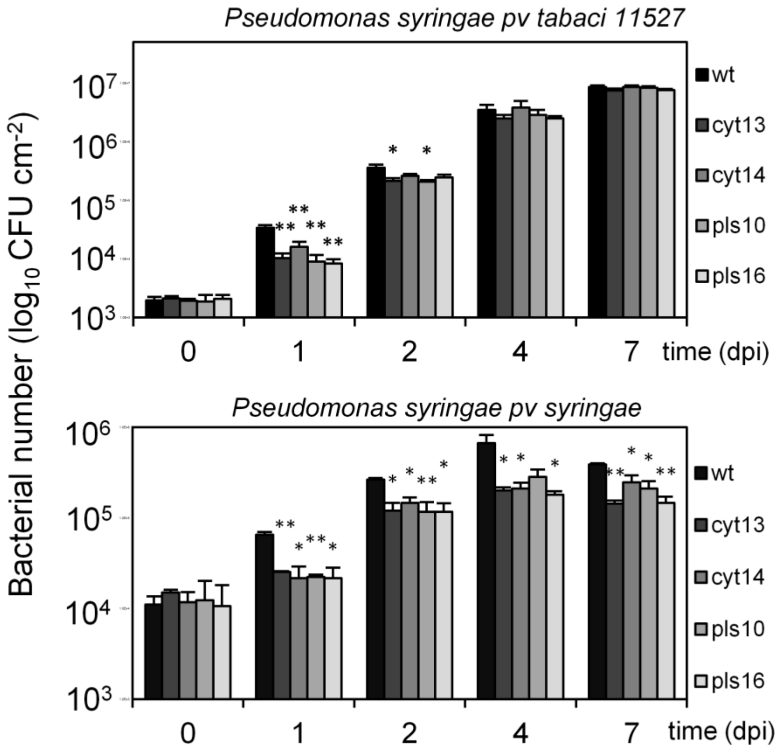

Time course of bacterial propagation in infected areas, inoculated with *Pst* strain 11527 (10^4 CFU ml^{-1}) and *Pss* ($5x10^5$ CFU ml^{-1})

Results represent mean values of four independent samples ± standard deviation.

Student t-test was employed to calculate significant difference between WT and HGLs (significant differences marked with asterisks * $p<0.05$, ** $p<0.01$). Experiments were repeated twice with similar results.

For further details see Fig. 8B.

Supplementary Fig. S6

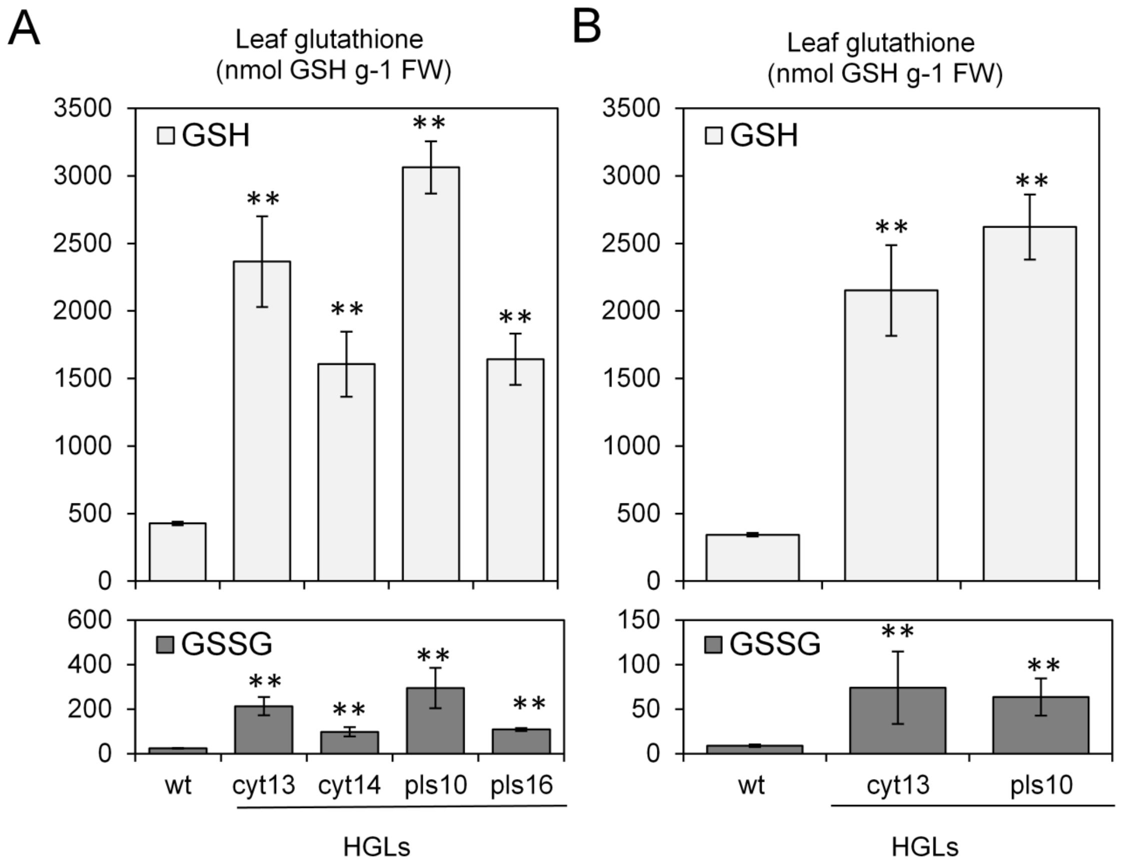

Glutathione content in tobacco WT and transgenic HGLs

Mean GSH and GSSG contents as determined by HPLC, measured in three plant individuals of each line used in experiments (A) for bacterial symptoms and propagation (see Fig. 8A,B), and (B) for HR-related conductivity and SA measurements (see Fig. 8C and Fig. 9A,B).

Results represent mean values of 3-4 independent samples ± standard deviation.

Student t-test was employed to calculate significant difference between WT and HGLs (significant differences marked with asterisks ** $p<0.01$).

Supplementary Fig. S7

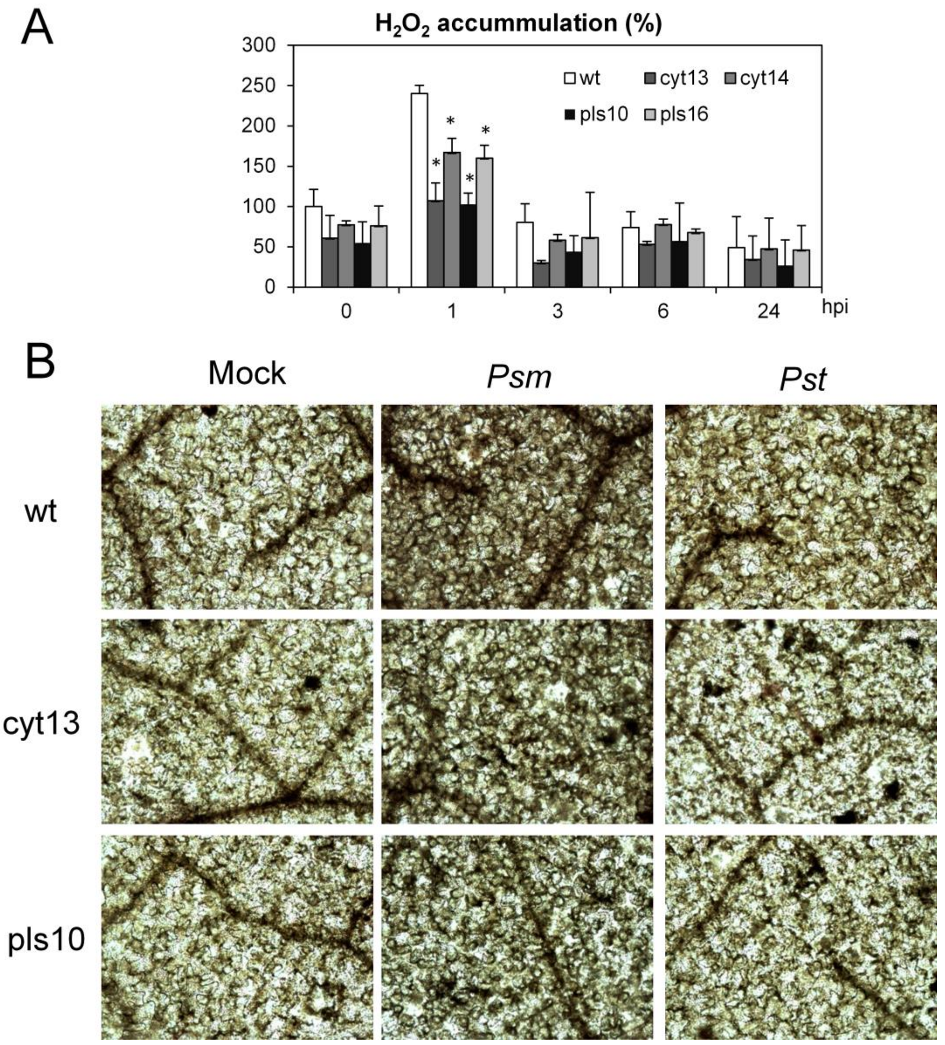

Hydrogen peroxide detection after infection with the non-adapted pathovar *Psm*

(A) Time course of H_2O_2 accumulation in infected tissue of WT and HGLs. Results represent mean values of three independent samples ± standard deviation. Student t-test was employed to calculate significant difference ($p<0.05$) between WT and HGL pls10 (significant differences marked with asterisks).

(B) DAB staining 24 hpi with $5x10^5$ CFU ml^{-1} bacteria; for mock treatment 10 mM $MgCl_2$ was applied.

Supplementary Table S1: List of primers used in the experiments

Gene	Gene name	Species	Accession No	Primer sequence
UBI	Ubiquitin	*N. tabacum*	U66264	5'-TGCAAGTCGGACGGGAAGTGGT-3’<br>5'-AGCCGTTTCCAGCTGTTGTTCCC-3’
L25 RP	Ribosomal protein L25	*N. tabacum*	L18908	5'CCCCTCACCACAGAGTCTGC-3’<br>5'AAGGGTGTTGTTGTCCTCAATCTT-3’
EF1α	Elongation factor 1α	*N. tabacum*	AF120093	5’-GGA CTG CCA CAC CTC CCA TGC3’<br>5’AGGAACCTGGGCTCTTTCGCA3’
PR1	Pathogenesis-related protein 1	*N. tabacum*	X12485	5'AGGCCGTTGAGATGTGGGTCG-'3<br>5'ACCGAGTTACGCCAAACCACCTG-'3
PR2	Pathogenesis-related protein 2	*N. tabacum*	M60460	5'TCTGAAAGTGGCTGGCCTTCTGA-'3<br>5'CCAGGTTTCTTTGGAGTTCCTGCCC-'3
PR4	Pathogenesis-related protein 4	*N. tabacum*	X60281	5'CCTCGAGGCCAAGATTCCTGTGG'3<br>5'AATCAAGCCCTCCATTGCTGCA-'3
PR5	Pathogenesis-related protein 5	*N. tabacum*	AF154636	5'TGGTGAGACTGGACTCCCAGAAGC-'3<br>5'ATGCCTTCTTTGCAGCAGCCT-'3
PR10	Pathogenesis-related protein 10	*N. tabacum*	AY055111.1	5'CATTGGTTGGAGGTGATGGATTGGT-'3<br>5'TGGAACCACCATCTGCTGACTGC-'3
NPR1	Non-expressor of pathogenesis-related genes 1	*N. tabacum*	AF480488	5'GGCGATGATTTGCGTATGAAGCTGT-'3<br>5'GCCGATGCTAGCCAGTGGGA-'3
ICS1	Isochorismate synthase	*N. tabacum*	AY740529	5'TGAGGGGGAGACTCCAGACTGA-'3<br>5'AGCCCGTGCATCTTCTGTAGGA-'3
SIPK	Salicylic acid Induced Protein kinase	*N. tabacum*	NTU94192	5'GCACATCCTTACCTGAACTCGCTCC-'3<br>5'TGTTCCTCCGTAAGGGCATGCT-'3
WIPK[1]	Wound Induced Protein Kinase	*N. bentamiana*	AB098729	5′-CCGATCTGCCCCGTTCCATCC-3′<br>5′-TCAGGATTCAGCGACAAAGCTTCC-3′
Ntf6[1]	Mitogen-activated protein kinase homolog	*N. bentamiana*	AB360634	5′-AAGGGGTTCCAACACATGAGGGG-3′<br>5′-GCCACGGCCGACAGGTTGAA-3′
Pti5[2]	PAMP-triggered immunity 5	*N. bentamiana*	SGN-U430823	5'CCTCCAAGTTTGAGCTCGGATAGT-'3<br>5'CCAAGAAATTCTCCATGCACTCTGTC-'3
CYP70D21[1]	Elicitor-inducible cytochrome P450	*N. bentamiana*	AF368376.2	5′-AAGGTCCACCGCACCATGTCCTTAGAG-3′<br>5′-AAGAATTCCTTGCCCCTTGAGTACTTGC-3′

cytRoGFP_F	5’GGGGACAAGTTTGTACAAAAAAGCAGGCTAGAGAGATGGCTCAAGAGTTTGTGAA3’
cytRoGFP_R	5’GGGGACCACTTTGTACAAGAAAGCTGGGTCCTATTACTTGTACAGCTCGTCCA3’

1 S**egonzac C, Feike D, Gimenez-Ibanez S, Hann DR, Zipfel C, Rathjen JP** (2011) Hierarchy and roles of pathogen-associated molecular pattern-induced responses in Nicotiana benthamiana. Plant Physiol **156:** 687-699

2 **Nguyen HP, Chakravarthy S, Velásquez AC, McLane HL, Zeng L, Nakayashiki H, Park D-H, Collmer A, Martin GB** (2010) Methods to Study PAMP-Triggered Immunity Using Tomato and Nicotiana benthamiana. Molecular Plant-Microbe Interactions **23:** 991-999
